# Three-dimensional wormhole with cosmic string effects on eigenvalue solution of non-relativistic quantum particles

**DOI:** 10.1038/s41598-023-40066-z

**Published:** 2023-08-10

**Authors:** Faizuddin Ahmed

**Affiliations:** Department of Physics, University of Science and Technology Meghalaya, Ri-Bhoi, Meghalaya 793101 India

**Keywords:** Quantum physics, General relativity and gravity

## Abstract

In this paper, we explore the quantum system of non-relativistic particles in a unique scenario: a circularly symmetric and static three-dimensional wormhole space-time accompanied by cosmic strings. We focus on a specific case where the redshift function $\varPhi (r)$ to be zero and defining the shape function as $A(r)=\frac{b}{r^2}$. After establishing this background space-time, we investigate the behavior of a harmonic oscillator within the same wormhole context. By doing so, we observe the effects of the cosmic string and wormhole throat radius on the eigenvalue solution of the oscillator’s eigenvalue problem. The primary finding is that these cosmic features lead to modifications in the energy spectrum and wave functions of the system, breaking the degeneracy of energy levels that would typically be present in a more conventional setting. As a particular case, we present the specific energy level $E_{1,\ell }$ and the corresponding wave function $\psi _{1,\ell }$, which are associated with the ground state of the quantum system. These results highlight the fascinating and unique properties of the harmonic oscillator in the background of a circularly symmetric, static wormhole space-time with cosmic strings.

## Introduction

The general theory of relativity is a profound scientific framework that establishes a fundamental connection between the curvature of space-time and the distribution of matter’s stress-energy^1^. When matter and energy are present, they cause the geometry of space-time to curve, and this curvature governs the dynamics of objects moving within it. This theory is exceptionally intricate, with its field equations consisting of ten non-linear differential equations. Finding exact solutions to these equations without imposing any symmetry or asymptotic conditions is extremely challenging.

To tackle this complexity, scientists often resort to employing both analytic and numerical approximations. Through these approximations, they can explore a wide range of fascinating physical phenomena, including gravitational lensing, the gravitational collapse of stars leading to the formation of black holes, the Big Bang theory, and the cosmic microwave background (CMB). By utilizing these approximation methods, researchers can gain valuable insights into the behavior of matter and energy in the presence of gravitational fields, unravelling the mysteries of some of the universe’s most enigmatic and awe-inspiring phenomena.

Numerous authors have successfully derived exact solutions to the field equations in different dimensions, including $(1+1)$, $(1+2)$, $(1+3)$, and higher dimensions. In four dimensions, some well-known solutions found in the literature encompass the Schwarzschild vacuum solution^2^, de-Sitter and anti-de Sitter space-times, Friedmann–Robertson–Walker (FRW) space-time, and the Kerr rotating solution^3^. However, there are other exact solutions that possess peculiar properties, such as the Gödel space-time^4^, the Som–Raychaudhri space-time^5^, Tipler’s rotating cylinder^6^, Gott time machine space-time^7^, and Ori time-machine space-time^8^, among others. These solutions exhibit characteristics like closed time-like curves (CTCs), closed time-like geodesics (CTGs), and closed null geodesics (CNGs), which violate causality conditions in general relativity and potentially allow for time travel. To address this issue and prevent the appearance of such peculiar properties in exact solutions of the field equations, S. Hawking postulates the Chronology Protection Conjecture (CPC)^9^. However, despite the conjecture, there is currently no rigorous proof available in the literature. Hence, the possibility of closed causal curves in certain space-time solutions cannot be easily discarded. It is worth noting that the presence of closed time-like curves in a space-time would lead to the concept of time-travel within the framework of relativity theory.

In the context of $(1+2)$-dimensional space-time, there exist several known solutions to the field equations, including those presented in Refs.^10,11^, solutions with string sources^12^, spinless BTZ space-time^13,14^, charged black hole solutions^15^, solutions with perfect fluid sources^16^, charged-free black hole solutions^17^, and multi-black hole geometries^18^. These solutions contribute to a better understanding of the gravitational dynamics in lower-dimensional space-time scenarios.

Apart from the known solutions of the field equations that exhibit closed causal curves, there are other exact solutions featuring curvature singularities, known as black hole space-times. A crucial defining characteristic is the presence of an event horizon that covers the curvature singularity, indicating the existence of a black hole; otherwise, the solution does not represent a black hole. Two well-known black hole solutions in four dimensions are the Schwarzschild solution^2^ and the Kerr solution^3^. In addition to black hole solutions, there are solutions of the field equations that violate the weak energy condition (WEC) and the null energy condition (NEC), creating wormhole space-times. However, a few wormhole space-time satisfies the weak and null energy condition. These solutions have gained significant attention in recent times due to their intriguing properties. Wormholes are theoretical structures that could potentially serve as shortcuts or narrow throats connecting two distinct regions of the universe. The possibility of traversing these wormholes has led to speculation about their potential as a tool for time travel. The concept of wormholes was independently introduced by Bronnikov^19^ and Ellis^20^ in four dimensions, resulting in the Ellis-Bronnikov wormhole model. Subsequently, Morris and Thorne proposed a traversable wormhole solution^21^ known as the Morris-Thorne wormhole model, which has garnered significant attention among researchers. Following these pioneering works, numerous wormhole space-times in four dimensions, with or without a cosmological constant, have been reported in the literature^22–29^. The study of these wormhole solutions continues to be an active area of research, as they present intriguing possibilities and implications within the framework of general relativity.

Indeed, efforts have been made to construct wormhole solutions in three-dimensional space-time as well. Several intriguing solutions have been proposed, each offering unique characteristics and implications within the context of general relativity. Some of the notable three-dimensional wormhole solutions include: traversable wormhole solution^30^, static and cyclic symmetric traversable wormhole^31^, traversable wormhole with a cosmological constant^25^, stable thin-shell wormhole^32^, stable charged thin-shell wormholes^33^, circular thin-shell wormholes^34^, traversable Lorentzian wormhole^35^. Each of these solutions contributes to a deeper understanding of the possibilities and implications of wormholes in three-dimensional space-time. The study of these solutions continues to be an active area of research, as they offer intriguing avenues for exploring exotic geometries and potential shortcuts in the fabric of the universe.

Topological defects arise as a consequence of spontaneous symmetry breaking in gauge theories during the phase transition in the early universe, as discussed in^36^. These defects are categorized into various types, including cosmic strings, domain walls, global monopoles, textures, and branes. The presence of topological defects significantly alters the geometric properties of the space-time being considered. Cosmic strings and global monopoles are two types of topological defects that have received extensive study in the realms of gravitation and cosmology, solid-state physics, and quantum mechanics. In quantum systems, the presence of topological defects induces changes in the behavior of quantum mechanical particles, thereby shifting the energy spectrum and wave functions of these particles, regardless of whether they are spin-zero, spin-half, or spin-one particles. In the domain of non-relativistic quantum systems, researchers have investigated the quantum motion of particles in the presence of topological defects such as cosmic strings^37^, and point-like global monopoles^38–42^. Some other investigations of the non-relativistic quantum systems in the background of the topological defects have been done in Refs.^43–50^. These studies provide valuable insights into the quantum dynamics of particles in the vicinity of topological defects, shedding light on the fascinating effects arising from the interplay of quantum mechanics and the underlying geometry of space-time.

Researchers have explored the effects of non-relativistic quantum mechanics on the harmonic oscillator problem within the context of topological defects. Notably, investigations have been carried out in scenarios such as: an elastic medium with spiral dislocation^51^, quantum revival time^52^, space-time with a distortion of a vertical line into a vertical spiral^53^, space-time with a screw dislocation subject to linear confining potential^54^, conical singularities space-time^38^, space-time with a linear topological defect^55^, in a point-like defect^56^, under non-inertial effects with a screw dislocation^57^, and in a topologically charged Ellis-Bronnikov-type wormhole^58^. These investigations offer valuable insights into the interplay between quantum mechanics and the presence of topological defects, enriching our understanding of the behavior of quantum systems in intriguing and non-trivial space-time backgrounds. The exploration of such effects holds promise for advancing our knowledge of quantum phenomena in diverse physical systems.

A circularly symmetric and static three-dimensional traversable wormhole space-time with cosmic string is described by the following line-element^30^

1 $$\begin{aligned} ds^2=-e^{\varPhi (r)}\,dt^2+\frac{dr^2}{\Big (1-\frac{A(r)}{r}\Big )}+\alpha ^2\,r^2\,d\phi ^2, \end{aligned}$$

where $\varPhi (r)$ is the red shift function, *A*(*r*) is the shape function and $\alpha <1$ is the cosmic string parameter. Noted that we introduce a cosmic string in this wormhole space-time by redefining the azimuthal angle $\phi$ in such a way that $\phi \rightarrow \phi '=\alpha \,\phi$. For a traversable wormhole, the shape function *A*(*r*) must satisfy flare-out condition, that is, $A(r)|_{r=r_0}=r_0$, and $A'(r)|_{r=r_0}<1$, where $r_0$ is the minimum global radius of the wormhole throat. This wormhole geometry to be asymptotically flat provided, we have the condition $\frac{A(r)}{r} \rightarrow 0$ and $\varPhi (r) \rightarrow 0$ at $r \rightarrow \infty$. The radial coordinate *r* has a range that increases from a minimum value at $r_0$, corresponding to the wormhole throat, to $\infty$, that is, $r \in [r_0, \infty )$ and other coordinates are $-\infty< t < +\infty$, $0 \le \phi < 2\,\pi$ with an angular deficit $\delta \phi =2\,\pi \,(1-\alpha )$. To avoid the presence of event horizons, $\varPhi (r)$ is imposed to be finite throughout the coordinate range. The presence of cosmic string changes the geometrical properties of a space-time under investigation.

For the above space-time , the non-zero components of the Einstein tensor are

2 $$\begin{aligned}{} & {} G^{t}_t=\frac{e^{\varPhi (r)}}{2\,r^3}\,\Big [A (r)-r\,A'(r)\Big ],\quad G^{r}_{r}=\frac{\varPhi '(r)}{2\,r}\,\Big (1-\frac{A(r)}{r}\Big ),\nonumber \\{} & {} G^{\phi }_{\phi }=\frac{1}{4\,r^2}\,\Big [\Big \{A(r)-r\,A'(r)+r\,\Big (r-A(r)\Big )\,\varPhi '(r)\Big \}\,\varPhi '(r)+2\,r\,\Big (r-A(r)\Big )\,\varPhi ''(r)\Big ]. \end{aligned}$$

Below, we discuss a special case by choosing the constant redshift function $\varPhi (r)$ and different form function *A*(*r*) as considered in Ref.^59^.

**Special case : constant redshift function**

As mentioned earlier, to prevent the formation of event horizons, the redshift function $\varPhi (r)$ should be finite throughout everywhere. A particular case which we are interested here is the solution with a constant redshift function, $\varPhi '(r)=0$. Without a loss of generality, we have considered $\varPhi (r)=0$. In that situation, time-component of the metric tensor for the space-time (1) is $g_{tt}=-1$. This specific case simplifies the field equations significantly, and provide particularly intriguing solution. The energy-momentum tensor is chosen to have the form $T^{\mu }_{\,\nu }=\text{ diag }(-\rho ,p_r,p_t)$, where $\rho$ represents the energy-density, $p_r$ represent the radial pressure, and $p_t$ represent the tangential pressure.

Under this case, that is, $\varPhi (r)=0$, one will find the non-zero energy density and zero pressure components, respectively given by

3 $$\begin{aligned} \rho =\frac{1}{2\,r^3}\,\Big [r\,A'(r)-A(r)\Big ]=R/2,\quad p_r=0=p_t. \end{aligned}$$

A pressure-less perfect fluid which violates the weak energy condition since $\frac{r\,A'(r)-A(r)}{A^2}<0$, the flaring-out condition. The Ricci scalar and the Kretschmann scalar curvatures for this case are given by

4 $$\begin{aligned} R=\frac{-A(r)+r\,A'(r)}{r^3},\quad \mathscr {K}=\frac{\Big [-A(r)+r\,A'(r)\Big ]^2}{r^6}=R^2. \end{aligned}$$

We consider next a few specific choices for the form function or the shape function *A*(*r*).

1. *Shape function*: $$A(r)=r_0$$
  For this case, the energy-density of the pressure-less perfect fluid, the Ricci scalar *R*, and the Kretschmann scalar $\mathscr {K}$ are given by
  5 $$\begin{aligned} \rho =-\frac{r_0}{2\,r^3},\quad R=-\frac{r_0}{r^3},\quad \mathscr {K}=\frac{r^{2}_0}{r^6}. \end{aligned}$$
  From above, we see that the perfect fluid violate violates the weak energy condition (WEC), $T_{\mu \nu }\,U^{\mu }\,U^{\nu }=\rho <0$ as well as the null energy condition (NEC), $T_{\mu \nu }\,k^{\mu }\,k^{\nu }=\rho \,U_{\mu }\,U_{\nu }\,k^{\mu }\,k^{\nu }<0$, where $U^{\mu }$ is the time-like vector and $k^{\mu }$ is a null vector. Furthermore, we see that all the physical quantities in (5) are finite at $r=r_0$ and vanishes for $r \rightarrow \infty$.
2. * Shape function*: $$A(r)=\frac{r^{2}_0}{r}$$
  For the specific case $A(r)=\frac{r^{2}_0}{r}$, the energy-density of pressure-less perfect fluid, the Ricci scalar *R*, and the Kretschmann scalar $\mathscr {K}$ are given by
  6 $$\begin{aligned} \rho =-\frac{r^{2}_0}{r^4},\quad R=-\frac{2\,r^{2}_0}{r^4},\quad \mathscr {K}=\frac{4\,r^{4}_0}{r^8}. \end{aligned}$$
  Here also, one can see that the perfect fluid violates the weak energy condition (WEC) and the null energy condition (NEC). All the physical quantities in (6) are finite at $r=r_0$ and vanishes for $r \rightarrow \infty$.
3. *Shape function*: $$A(r)=r_0\,\Big [1+\gamma \,\Big (1-\frac{r_0}{r}\Big )\Big ]$$
  Lastly, we choose the following form function
  7 $$\begin{aligned} A(r)=r_0\,\Big [1+\gamma \,\Big (1-\frac{r_0}{r}\Big )\Big ], \end{aligned}$$
  where $0< \gamma < 1$ otherwise the flare-out condition will not satisfy.
  In that case, the energy-density of the pressure-less perfect fluid, the Ricci scalar *R*, and the Kretschmann scalar $\mathscr {K}$ are given by
  8 $$\begin{aligned} \rho =\frac{r_0}{2\,r^4}\Big [2\,\gamma \,r_0-(1+\gamma )\,r\Big ],\quad R=\frac{r_0}{r^4}\Big [2\,\gamma \,r_0-(1+\gamma )\,r\Big ],\quad \mathscr {K}=\frac{r^{2}_0}{r^8}\Big [-2\,\gamma \,r_0+(1+\gamma )\,r\Big ]^2. \end{aligned}$$
  At $r=r_0$, the wormhole throat radius, the energy-density given by
  9 $$\begin{aligned} \rho |_{r=r_0}=\frac{(\gamma -1)}{2\,r^2_{0}}<0 \quad (0< \gamma < 1) \end{aligned}$$
  violate the energy conditions for the given range of $\gamma$. All physical quantities given in (8) are finite at $r=r_0$ and vanishes for $r \rightarrow \infty$.
  In this work, we consider the form function or shape function of the second kind given by $A(r)=\frac{b^2}{r}$, where $b>0$. Therefore, circularly symmetric and a static $(1+2)$-dimensional traversable wormhole space-time with a cosmic string is given by the following line-element
  10 $$\begin{aligned} ds^2=-dt^2+\frac{r^2\,dr^2}{(r^2-b^2)}+\alpha ^2\,r^2\,d\phi ^2, \end{aligned}$$
  where $b=const=r_0$ is the wormhole throat radius. One can see that if we choose $b \rightarrow 0$, the line-element (11) becomes a cosmic string space-time in three dimensions. Finally, introducing a new coordinate $r^2=(x^2+b^2)$ into this space-time (11) covering the whole wormhole regions, one will obtain the following line-element
  11 $$\begin{aligned} ds^2=-dt^2+dx^2+\alpha ^2\,(x^2+b^2)\,d\phi ^2=-dt^2+g_{ij}\,dx^{i}\,dx^{j}, \end{aligned}$$
  where $x^1=x, x^2=\phi$. The ranges of the different coordinates are $-\infty< t < \infty$, $0 \le \phi < 2\,\pi$, and the coordinate *x* runs from $-\infty$ to $+\infty$, where $x=0$ represents the wormhole throat. The non-zero covariant and contravariant components of the spatial metric tensor $g_{ij}$ are given by
  12 $$\begin{aligned} g_{xx}=1=g^{xx},\quad g_{\phi \phi }=\alpha ^2\,(x^2+b^2)=\frac{1}{g^{\phi \phi }} \end{aligned}$$
  with its determinant $g=|g_{ij}|=\alpha ^2\,(x^2+a^2)$.

This research work focuses on the investigation of quantum system of non-relativistic particles within the context of a circularly symmetric and static traversable wormhole space-time featuring cosmic strings. The primary objective is to explore how the presence of cosmic strings and the wormhole throat radius influence the solution of time-independent eigenvalue equation. To achieve this, the study begins by analyzing the quantum behavior of particles in the given wormhole background. The wave equation is solved for this specific scenario, allowing for the determination of the energy levels and corresponding wave functions. Subsequently, we investigate the harmonic oscillator problem within the same wormhole background, considering its impact on the eigenvalue solutions. The key findings reveal that the presence of cosmic strings and the wormhole throat radius significantly modify the energy levels and wave functions of the quantum particles. The results obtained demonstrate clear shifts in the quantum properties of the system under the influence of these factors. Overall, this research uncovers the intricate interplay between quantum mechanics and the geometrical properties of a traversable wormhole with cosmic strings, shedding light on how such exotic features can shape the quantum dynamics of particles. The findings contribute to a deeper understanding of the behavior of quantum systems in non-trivial space-time backgrounds and may have implications for various areas of theoretical physics. So far author’s concern, this is the first investigation of the non-relativistic quantum system in the background of three-dimensional wormhole space-time with a cosmic string.

The paper is structured as follows: In “Non-relativistic quantum particles in three-dimensional wormhole with a cosmic string” section, we derive the time-independent wave equation governing the behavior of non-relativistic particles in a circularly symmetric wormhole space-time background. The wave equation is then solved using the Heun function, which allows us to gain insights into the quantum dynamics of particles within this unique wormhole configuration. Moving on to “Harmonic oscillator in Three-dimensional wormhole with a cosmic string” section, we explore the harmonic oscillator problem within the same wormhole background. The wave equation for the harmonic oscillator is solved using the same method employed in “Non-relativistic quantum particles in three-dimensional wormhole with a cosmic string” section. This investigation provides a deeper understanding of how the harmonic oscillator behaves in the presence of cosmic strings and the wormhole throat radius. Finally, in “Conclusions” section, we present our conclusions based on the findings from the previous sections. We summarize the key results and discuss their implications in the context of the circularly symmetric and static traversable wormhole space-time with cosmic strings. Throughout the entire analysis, we adopt a system of units in which the fundamental constants *c*, $\hbar$, and *G* are set to unity, simplifying the mathematical expressions and facilitating a more concise representation of the results.

## Non-relativistic quantum particles in three-dimensional wormhole with a cosmic string

In this section, we delve into the quantum system of non-relativistic particles using the time-independent Schrödinger wave equation in the presence of a circularly symmetric and static wormhole background with a cosmic string. To initiate our investigation, we start by defining the Hamiltonian operator for a non-relativistic particle, which is given as follows^38,38–41,56,57^:

13 $$\begin{aligned} \hat{H}=-\frac{1}{2\,M}\,\frac{1}{\sqrt{g}}\,\partial _{i}\,\Big (\sqrt{g}\,g^{ij}\,\partial _{j}\Big ), \end{aligned}$$

where *M* is the rest mass of the particles, $g=|g_{ij}|$ is the determinant of the metric tensor $g_{ij}$ with $g^{ij}$ its inverse. This Hamiltonian operator is a fundamental quantity in quantum mechanics, and its role in describing the dynamics of non-relativistic particles in the presence of the considered wormhole space-time with cosmic strings is pivotal to our study. We will use this operator to derive the Schrödinger wave equation and explore its solutions, shedding light on the intriguing behavior of quantum systems in this exotic space-time background.

Expressing the Hamiltonian (13) in the space-time background (11) and using (12), we obtain

14 $$\begin{aligned} \hat{H}=-\frac{1}{2\,M}\,\Bigg [\frac{\partial ^2}{\partial x^2}+\frac{x}{(x^2+b^2)}\,\frac{\partial }{\partial x}+\frac{1}{\alpha ^2\,(x^2+b^2)}\frac{\partial ^2}{\partial \phi ^2} \Bigg ]. \end{aligned}$$

The eigenvalue equation of the non-relativistic particles is given by^38,56^

15 $$\begin{aligned} \hat{H}\,\varPsi (x, \phi )=E\,\varPsi (x, \phi ), \end{aligned}$$

where *E* is the particles energy eigenvalue.

The wave function can be expressed in terms of the function $\psi (x)$ as follows

16 $$\begin{aligned} \varPsi (x, \phi )=e^{i\,\ell \,\phi }\,\psi (x), \end{aligned}$$

where $\ell =0\,\pm \,1,\pm \,2,...$ are the eigenvalues of the orbital quantum number associated with the operator $-i\,\hat{\partial }_{\phi }$.

Thereby, substituting (14) into the Eq. (15) and using the wave function (16), we obtain the following differential equation

17 $$\begin{aligned} \psi ''(x)+\frac{x}{(x^2+b^2)}\,\psi '(x)+\Bigg [\sigma ^2-\frac{\ell ^2_{0}}{(x^2+b^2)}\Bigg ]\,\psi (x)=0, \end{aligned}$$

where we have set

18 $$\begin{aligned} \ell _0=\frac{|\ell |}{\alpha }\quad ,\quad \sigma ^2=2\,M\,E. \end{aligned}$$

Here, we see that the orbital quantum number gets shifted or modified, that is, $\ell \rightarrow \ell _0=\frac{|\ell |}{\alpha }$ by the cosmic string parameter $\alpha$.

Introducing a new variable via $x^2=-b^2\,u$ in the Eq. (17), we obtain the following differential equation

19 $$\begin{aligned} \psi ''(u)+\Bigg [\frac{1/2}{u}+\frac{1/2}{u-1} \Bigg ]\,\psi '(u)+\Bigg [\frac{(\ell ^2_{0}-\sigma ^2\,b^2)/4}{u}+\frac{(-\ell ^2_{0}/4)}{u-1} \Bigg ]\,\psi (u)=0. \end{aligned}$$

Equation (19) is the confluent Heun equation form^53,60–62^ with $\psi (u)$ is the confluent Heun function given by

20 $$\begin{aligned} \psi (u)=H_{c}\left( 0, -\frac{1}{2}, -\frac{1}{2}, -\frac{\sigma ^2\,b^2}{4}, \frac{\sigma ^2\,b^2}{4}+\frac{3}{8}-\frac{\ell ^2_{0}}{4}; u\right) . \end{aligned}$$

To obtain bound-states solution of the quantum system, let us consider the function $\psi (u)$ to be a power series solution around the origin^63^ given by

21 $$\begin{aligned} \psi (u)=\sum _{i=0}^{\infty }\,f_{i}\,u^{i}. \end{aligned}$$

Substituting this power series (21) in the Eq. (19), we obtain the following recurrence relation

22 $$\begin{aligned} f_{k+2}=\frac{1}{2\,(k+2)\,(2\,k+3)}\Bigg [\Big \{4\,(k+1)^2+\sigma ^2\,b^2-\ell ^2_{0}\Big \}\,f_{k+1}-\sigma ^2\,b^2\,f_{k}\Bigg ] \end{aligned}$$

with the coefficient

23 $$\begin{aligned} f_1=\frac{1}{2}\,(\sigma ^2\,b^2-\ell ^2_{0})\,f_0. \end{aligned}$$

One can see from the recurrence relation that a closed or compact expression of the energy eigenvalue may not be possible by setting $f_{n+1}=0$ and $\sigma ^2\,b^2=0$. Therefore, we follow another procedure by setting $k=(n-1)$ where the coefficient $f_{n+1}=0$. Therefore, using this this condition in the recurrence relation (22), we obtain

24 $$\begin{aligned} f_{n}=\frac{\sigma ^2\,b^2}{\Big [4\,n^2+\sigma ^2\,b^2-\ell ^2_{0}\Big ]}\,f_{n-1}. \end{aligned}$$

Now, one can find the individual energy levels and wave functions of the quantum mechanical particles one by one by setting $n=1$ and others are in the same way. The ground state or lowest state of the quantum system is defined by $n=1$, and thus, from the relation (24), we obtain

25 $$\begin{aligned} f_1=\frac{\sigma ^2\,b^2}{(4+\sigma ^2\,b^2-\ell ^2_{0})}\,f_{0}. \end{aligned}$$

Comparing Eqs. (23) and (25), we obtain the following ground state energy level given by

26 $$\begin{aligned} E^{\pm }_{1,\ell }=\frac{1}{2\,M\,b^2}\,\Bigg [-1+\frac{|\ell |}{\alpha }\pm \sqrt{1+2\,\frac{|\ell |}{\alpha }}\Bigg ]. \end{aligned}$$

And that the corresponding wave function will be

27 $$\begin{aligned} \varPsi ^{\pm }_{1,\ell } (u, \phi )=e^{i\,\ell \,\phi }\,\Bigg [\Big (1-\frac{u}{2}\Big )\pm \frac{u}{2}\sqrt{1+2\,\frac{|\ell |}{\alpha }}\Bigg ]\,f_0. \end{aligned}$$

**Figure 1:**
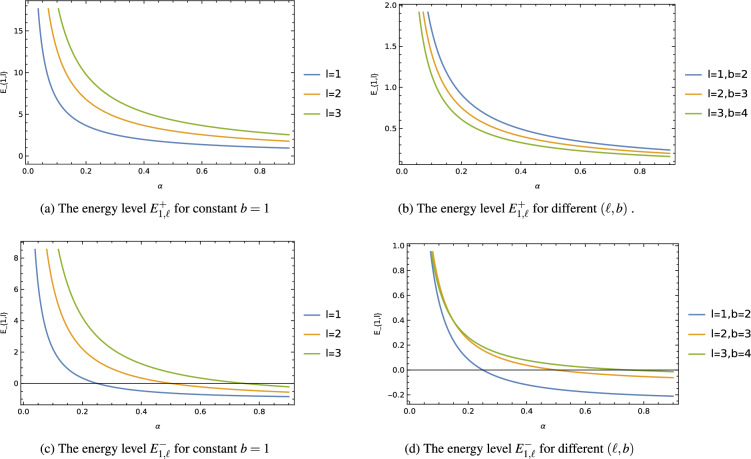
The ground state energy level $E^{\pm }_{1,\ell }$ with cosmic string parameter $\alpha$ for different values of $(\ell ,b)$.

**Figure 2:**
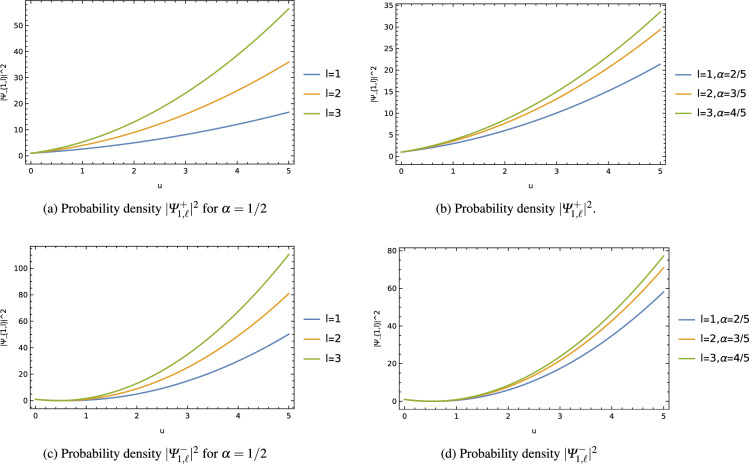
Probability density $|\varPsi ^{\pm }_{1,\ell }|^2$ for different values of $(\ell ,\alpha )$.

Equation (26) represents the ground state energy level, while Eq. (27) corresponds to the corresponding wave function of non-relativistic particles in a circularly symmetric and static wormhole space-time with cosmic strings. By following a similar approach, one can obtain other state energy levels and wave functions for the mode $n \ge 2$. It is evident from Eq. (26) that the lowest state energy level $E^{\pm }{1,\ell }$ and wave function $\psi ^{\pm }{1,\ell }$ are influenced by the cosmic string parameter $\alpha$ and the wormhole throat radius $b=const$. The presence of this cosmic string parameter in the quantum system effectively breaks the degeneracy of the energy levels and leads to a shift in their values.

To better understand this influence, graphs were generated to illustrate the impact of the cosmic string on the energy levels $E^{\pm }{1,\ell }$ and the probability density $|\varPsi ^{\pm }{1,\ell }|^2$. These graphs were plotted for different values of the orbital quantum number *l* and the wormhole throat radius *b*. In Fig. 1, it is observed that the energy level $E^{\pm }_{1,\ell }$ gradually decreases as the cosmic string parameter $\alpha$ increases up to certain values, after which it reaches a saturation point with further increments of this parameter. Moreover, the decreasing energy level is found to be more significantly shifted with increasing orbital quantum number $\ell$. In Fig. 2, the probability density of the non-relativistic particles steadily increases, and this increment is further enhanced with increasing values of the orbital quantum number $\ell$ and the cosmic string parameter $\alpha$.

These results provide valuable insights into how the presence of cosmic strings affects the energy levels and wave functions of the quantum system, offering a deeper understanding of the interplay between the cosmic string parameter, the wormhole throat radius, and the quantum behavior of particles within this intriguing wormhole space-time background.

For $\alpha \rightarrow 1$, the ground state energy eigenvalue of the non-relativistic particle becomes

28 $$\begin{aligned} E_{1,\ell }=\frac{1}{M\,b^2}\,\Big (-1+|\ell | \pm \sqrt{1+2\,|\ell |}\Big ). \end{aligned}$$

The corresponding radial wave function will be

29 $$\begin{aligned} \varPsi ^{\pm }_{1,\ell } (u, \phi )=e^{i\,\ell \,\phi }\,\Bigg [\Big (1-\frac{u}{2}\Big )\pm \frac{u}{2}\sqrt{1+2\,|\ell |}\Bigg ]\,f_0. \end{aligned}$$

Equation (28) is the ground state energy level and Eq. (29) is the corresponding wave function of the non-relativistic particles in a circularly symmetric and static $(1+2)$-dimensional wormhole space-time background without any cosmic string effects.

## Harmonic oscillator in Three-dimensional wormhole with a cosmic string

In this section, we delve into the quantum system of non-relativistic particles interacting harmonically within a wormhole space-time background featuring cosmic strings [as described by Eq. (10)]. In other words, we explore the harmonic oscillator problem within this unique space-time configuration. To achieve this, we consider the Hamiltonian operator for a harmonic oscillator, which is given as follows^38,56,58^:

30 $$\begin{aligned} \hat{H}_{osc}=-\frac{1}{2\,M}\,\frac{1}{\sqrt{g}}\,\partial _{i}\,\Big (\sqrt{g}\,g^{ij}\,\partial _{j}\Big )+\frac{1}{2}\,M\,\omega ^2\,x^2, \end{aligned}$$

where $\omega$ is the oscillator frequency and other physical entities are mentioned earlier. This Hamiltonian operator governs the behavior of the harmonic oscillator in the given wormhole space-time background with cosmic strings. By solving the associated Schrödinger wave equation and analyzing its solutions, we gain valuable insights into the quantum behavior of the harmonic oscillator within this intriguing and exotic space-time setting. This investigation provides a deeper understanding of the interplay between quantum mechanics and the geometric properties of the wormhole space-time with cosmic strings.

Expressing Eq. (30) in the space-time background (11) and using (12), we obtain the following equation

31 $$\begin{aligned} \hat{H}_{osc}=-\frac{1}{2\,M}\,\Bigg [\frac{\partial ^2}{\partial x^2}+\frac{x}{(x^2+b^2)}\,\frac{\partial }{\partial x}+\frac{1}{\alpha ^2\,(x^2+b^2)}\,\frac{\partial ^2}{\partial \phi ^2}\Bigg ]+\frac{1}{2}\,M\,\omega ^2\,x^2. \end{aligned}$$

The energy eigenvalue equation of a harmonic oscillator is given by

32 $$\begin{aligned} \hat{H}_{osc}\,\varPsi (x, \theta , \phi )=E_{osc}\,\varPsi (x, \theta , \phi ), \end{aligned}$$

where $E_{osc}$ is the energy eigenvalue of the oscillator field.

Thereby, substituting Eq. (31) in the Eq. (32) and using the wave function (16), we obtain the following differential equation

33 $$\begin{aligned} \psi ''(x)+\frac{x}{(x^2+b^2)}\psi '(x)+\Big [\sigma ^2-M^2\,\omega ^2\,x^2-\frac{\ell ^2_{0}}{(x^2+b^2)}\Big ]\psi (x)=0, \end{aligned}$$

where $\sigma ^2, \ell ^2_{0}$ are defined earlier.

The requirement of the wave function $\psi$ is that it must be finite and regular everywhere for $x \rightarrow 0$ and $x \rightarrow \pm \,\infty$. Let us suppose a possible solution to the equation (33) given by

34 $$\begin{aligned} \psi (x)=exp\left( -\frac{1}{2}\,M\,\omega \,x^2\right) \,H(x), \end{aligned}$$

where *H*(*x*) is an unknown function.

Thereby, substituting solution (34) in the Eq. (33), we obtain the following differential equation

35 $$\begin{aligned} H''(x)+\left[ \frac{x}{x^2+b^2}-2\,M\,\omega \,x\right] \,H'(x)+\Bigg [\lambda ^2-\frac{j^2}{x^2+b^2} \Bigg ]\,H(x)=0, \end{aligned}$$

where we have set the parameters

36 $$\begin{aligned} \lambda ^2=\sigma ^2-2\,M\,\omega \quad ,\quad j^2=\ell ^2_{0}-M\,\omega \,b^2. \end{aligned}$$

Introducing a new variable via $s=-\frac{x^2}{b^2}$ in the Eq. (35), we obtain the following differential equation

37 $$\begin{aligned} H''(s)+\Big [M\,\omega \,b^2+\frac{1/2}{s}+\frac{1/2}{s-1}\Big ]\,H'(s)+\Bigg [\frac{(j^2-b^2\,\lambda ^2)/4}{s}+\frac{(-j^2/4)}{(s-1)}\Bigg ]\,H(s)=0. \end{aligned}$$

Equation (37) is the confluent Heun equation^53,60–62^ and *H*(*s*) is the confluent Heun function given by

38 $$\begin{aligned} H(s)=H_{c}\Big (M\,\omega \,b^2, -\frac{1}{2}, -\frac{1}{2}, -\frac{b^2\,\sigma ^2}{4},\frac{b^2\,\sigma ^2}{4}+\frac{3}{8}-\frac{\ell ^2_{0}}{4}; s\Big ). \end{aligned}$$

As stated earlier, to obtain bound-states solution of the harmonic oscillator, we must consider the Heun function *H*(*s*) to be a power series solution around the origin^63^ given by

39 $$\begin{aligned} H(s)=\sum _{i=0}^{\infty }\,d_{i}\,s^{i}. \end{aligned}$$

Substituting this power series (39) in the Eq. (37), we obtain the following recurrence relation

40 $$\begin{aligned} d_{k+2}=\frac{1}{2\,(k+2)(2\,k+3)}\Bigg [\Bigg \{4\,(k+1)(k+1-M\,\omega \,b^2)+b^2\,\lambda ^2-j^2\Bigg \}\,d_{k+1}-(b^2\,\lambda ^2-4\,M\,\omega \,b^2\,k)\,d_{k}\Bigg ]. \end{aligned}$$

with the coefficient

41 $$\begin{aligned} d_{1}=\frac{(b^2\,\lambda ^2-j^2)}{2}\,d_{0}. \end{aligned}$$

As mentioned earlier, finding a closed expression for the bound-state energy levels of the harmonic oscillator using the Heun function is not always possible. Instead, we will follow a procedure to obtain the individual energy levels and wave functions one by one. Let us consider a specific case where we set $k = (n-1)$, leading to the coefficient $d_{n+1}$ being equal to zero. This choice ensures that the Heun function $H(s) = (d_0 + d_1s + \ldots + d_{n}s^{n})$ becomes a finite-degree polynomial, simplifying the expression. Consequently, the wave function $\varPsi$ will be regular everywhere, avoiding any singularities. Thereby, setting $k=(n-1)$ and $d_{n+1}=0$, from the relation (40) we obtain

42 $$\begin{aligned} d_{n}=\frac{b^2\,\lambda ^2-4\,(n-1)\,M\,\omega \,b^2}{\Big [4\,n\,(n-M\,\omega \,b^2)-j^2+b^2\,\lambda ^2)\Big ]}\,d_{n-1}. \end{aligned}$$

As a particular case, let’s consider the mode $n=1$, which corresponds to the ground state or the lowest energy state of the quantum system. By setting $n=1$ in the Heun function expression, we can obtain the energy and wave function for the ground state of the harmonic oscillator. For the mode $n=1$ that corresponds to the ground state or the lowest state of the quantum system, from relation (42) we obtain

43 $$\begin{aligned} d_{1}=\frac{b^2\,\lambda ^2}{\Big [4-4\,M\,\omega \,b^2-j^2+b^2\,\lambda ^2\Big ]}\,d_{0}. \end{aligned}$$

**Figure 3:**
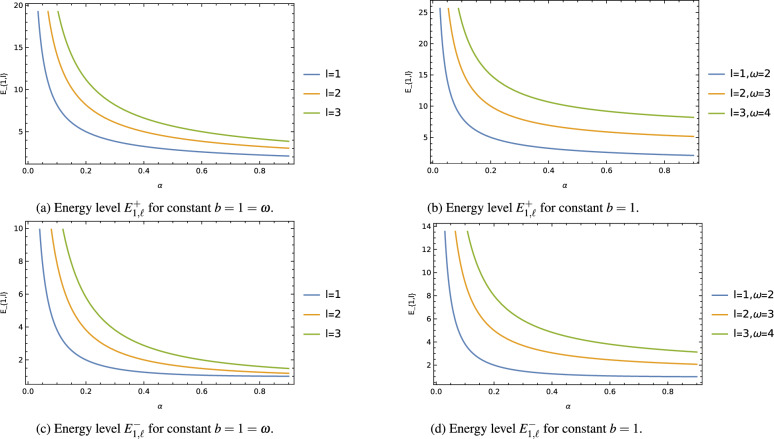
The ground state energy level $E^{\pm }_{1,\ell }$ with cosmic string parameter $\alpha$.

**Figure 4:**
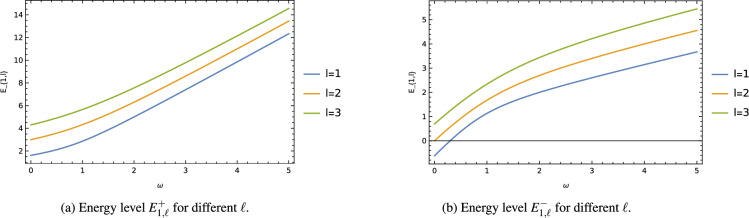
The energy level $E^{\pm }_{1,\ell }$ with oscillator frequency $\omega$ for different $\ell$ keeping fixed $b=1$, $\alpha =1/2$.

Thereby, comparing Eqs. (41) and (43), we obtain the following energy expression

44 $$\begin{aligned} E^{\pm }_{1,\ell }=\frac{3\,\omega }{2}+\frac{1}{2\,M\,b^2}\,\Bigg [-1+\frac{|\ell |}{\alpha }\pm \sqrt{1+2\,\frac{|\ell |}{\alpha }+4\,M\,\omega \,b^2\,\Big (M\,\omega \,b^2-\frac{3}{2}\Big )}\Bigg ], \end{aligned}$$

And that the corresponding wave function will be

45 $$\begin{aligned} \psi ^{\pm }_{1,\ell } (s)=exp\left( -\frac{1}{2}\,M\,\omega \,s^2\right) \Bigg [\Big (1-\frac{s}{2}\Big )+\Bigg \{M\,\omega \,b^2\pm \frac{1}{2}\sqrt{1+2\,\frac{|\ell |}{\alpha }+4\,M\,\omega \,b^2\,\Big (M\,\omega \,b^2-\frac{3}{2}\Big )}\Bigg \}s\Bigg ]d_0.\quad \quad \end{aligned}$$

Equation (44) represents the ground state energy level, while Eq. (45) corresponds to the corresponding wave function of the harmonic oscillator in a circularly symmetric and static wormhole space-time with cosmic strings in three dimensions. By employing a similar procedure, one can obtain other state energy levels and wave functions for the mode $n \ge 2$. It is important to note that when the oscillator frequency $\omega$ tends to zero ($\omega \rightarrow 0$), the eigenvalue solution derived in this section reduces to the results obtained in the previous section by Eqs. (26)–(27). This limiting case provides a connection between the harmonic oscillator in the wormhole space-time with cosmic strings and the harmonic oscillator in a standard space-time without the presence of cosmic strings. The obtained solutions offer valuable insights into the quantum behavior of the harmonic oscillator in the exotic background of a traversable wormhole with cosmic strings. Understanding how the oscillator behaves in such unique space-time configurations is crucial for exploring the effects of topological defects on quantum systems and their implications for various branches of physics. As shown in Eqs. (44)–(45), the energy levels and wave functions of the harmonic oscillator are significantly influenced by the cosmic string parameter $\alpha$ and the wormhole throat radius $b=const$. The presence of the cosmic string introduces shifts in the eigenvalue solutions of the harmonic oscillator, effectively breaking the degeneracy of the energy levels. The result presented in this section is completely different from those results obtained in the previous work^58^ which was done in a four-dimensional wormhole metric background with global monopole.

To better understand this influence, graphs were generated to illustrate the impact of the cosmic string parameter $\alpha$ and the oscillator frequency $\omega$ on the energy levels $E^{\pm }_{1,\ell }$ for various values of the other parameters. Figure 3 demonstrates that the energy level $E^{\pm }_{1,\ell }$ gradually decreases as the cosmic string parameter $\alpha$ increases, reaching a saturation point for certain values of $\alpha$. Additionally, the decreasing energy level experiences a greater shift with increasing values of the orbital quantum number *l* and the oscillator frequency $\omega$. In Figure 4, it is observed that the energy level $E^{\pm }_{1,\ell }$ increases almost linearly with the oscillator frequency $\omega$. Furthermore, this increasing energy level experiences a greater shift with increasing values of the orbital quantum number *l*, while keeping the wormhole throat radius $b=const$ and the cosmic string parameter $\alpha$ fixed.

These graphical results offer valuable insights into the behavior of the harmonic oscillator in the presence of cosmic strings within the exotic background of a traversable wormhole space-time. The shifts in the energy levels due to the cosmic string parameter and oscillator frequency provide important information about the effects of topological defects on the quantum dynamics of the harmonic oscillator. This understanding is essential for gaining a deeper knowledge of the interplay between quantum mechanics and the geometrical properties of space-time with cosmic strings and wormholes.

For $\alpha \rightarrow 1$, the ground state energy eigenvalue of a harmonic oscillator will become

46 $$\begin{aligned} E^{\pm }_{1,\ell }=\frac{3\omega }{2}+\frac{1}{M\,b^2}\Bigg [-1+|\ell |\pm \sqrt{1+2\,|\ell |+4\,M\,\omega \,b^2\,\Big (M\,\omega \,b^2-\frac{3}{2}\Big )}\Bigg ]. \end{aligned}$$

And that the corresponding wave function will be

47 $$\begin{aligned} \psi ^{\pm }_{1,\ell } (s)=exp\left( -\frac{1}{2}\,M\,\omega \,s^2\right) \Bigg [\Big (1-\frac{s}{2}\Big )+\Bigg \{M\,\omega \,b^2\pm \frac{1}{2}\sqrt{1+2\,|\ell |+4\,M\,\omega \,b^2\,\Big (M\,\omega \,b^2-\frac{3}{2}\Big )}\Bigg \}s\Bigg ]d_0. \end{aligned}$$

Equation (46) is the ground state energy level and Eq. (47) is the corresponding wave function of the harmonic oscillator in a circularly symmetric and static $(1+2)$-dimensional wormhole space-time background without any topological defects.

## Conclusions

The harmonic oscillator basis offers a formulation that treats momenta and coordinates equally, allowing for the incorporation of both long- and short-range interactions. This unique feature makes it particularly advantageous in nuclear-structure theory as it retains all symmetries of atomic nuclei while providing an approximate mean-field description related to the nuclear shell model. As a result, it becomes a valuable model for studying the behavior of atomic nuclei and their interactions.

The harmonic oscillator potential is an exact solvable potential model in quantum mechanics, making it of great interest and significance in various branches of physics and chemistry. Its applications span across different areas, providing valuable insights into the behavior of particles and systems in diverse physical and chemical contexts. Numerous researchers have attempted to map the free-particle Schrödinger equation to that of the Schrödinger equation for the harmonic potential. This mapping aims to explore the similarities and connections between these two different scenarios, potentially revealing important relationships and facilitating a deeper understanding of quantum systems under the influence of the harmonic potential. Overall, the harmonic oscillator basis is a versatile and fascinating model that not only exhibits exact quantum mechanical potential but also finds widespread applications across a multitude of scientific disciplines. Its ability to unify long- and short-range interactions in a coherent manner makes it an invaluable tool for investigating complex systems and phenomena.

In this study, we have thoroughly investigated the quantum system of non-relativistic particles within a wormhole background featuring topological defects caused by a cosmic string. Specifically, we have considered a circularly symmetric and static $(1+2)$-dimensional space-time with cosmic strings. By deriving the radial equation of the Schrödinger wave equation and converting it into the confluent Heun differential equation form, we were able to obtain exact solutions for the ground state energy level $E_{1,\ell }$ and wave function $\psi _{1,\ell }$ as particular cases, with similar procedures applicable for higher mode solutions.

Throughout our analysis, we have observed that the presence of the cosmic string and the wormhole throat radius significantly influence the energy levels and wave functions of the non-relativistic particles, resulting in their modifications. Graphs were generated to illustrate these influences, depicting how the ground state energy level $E_{1,\ell }$ varies with the cosmic string parameter (fig. 1) and how the probability density of the wave function changes (fig. 2) for different values of the orbital quantum number $\ell$ and the wormhole throat radius *b*.

Moreover, we have explored the harmonic oscillator problem within the same wormhole background, incorporating the cosmic string effect. By solving the radial wave equation, we presented the ground state energy level $E_{1,\ell }$ and wave function $\psi _{1,\ell }$ of the harmonic oscillator as particular cases. Similar to the non-relativistic particles, the energy levels and wave functions of the harmonic oscillator are influenced by the cosmic string and wormhole throat radius, resulting in modifications to their behavior. Additionally, the presence of the cosmic string leads to the breaking of degeneracy in the energy levels of the quantum mechanical particles. Graphs were also generated to illustrate the effect of the cosmic string parameter (Fig. 3) and the oscillator frequency (Fig. 4) on the ground state energy level $E_{1,\ell }$ for different values of the orbital quantum number $\ell$ and the wormhole throat radius *b*.

This comprehensive analysis provides valuable insights into the interplay between topological defects, cosmic strings, and wormholes, and their effects on the quantum dynamics of particles and the harmonic oscillator. The results shed light on the intricate behavior of quantum systems in exotic space-time backgrounds, advancing our understanding of the fundamental principles governing the universe.

### Supplementary Information

Supplementary Information.

## Data Availability

All data generated or analysed during this study are included in this published article.
