# Supplementary material for: Three-dimensional wormhole with cosmic string effects on eigenvalue solution of non-relativistic quantum particles
Source: Sci Rep. 2023 Aug 10;13:12953. doi: 10.1038/s41598-023-40066-z (PMC10415272; doi:10.1038/s41598-023-40066-z)
Supplement: Supplementary file 1 — Supplementary Information. [file 41598_2023_40066_MOESM1_ESM.pdf]

APPENDIX A: CIRCULARLY SYMMETRIC AND STATIC $(1+2)$-DIMENSIONAL WORMHOLE SPACE-TIME WITH COSMIC STRINGS

The original circularly symmetric and static wormhole metric is given by [30]

$$ds^2 = -e^{2\,\Phi(r)}\,dt^2 + \frac{dr^2}{\left(1-\frac{A(r)}{r}\right)} + r^2\,d\phi^2. \tag{A.1}$$

Setting the redshift function $\Phi(r)=0$ and shape function $A(r)=\frac{b^2}{r}$, we obtain the following metric

$$ds^2 = -dt^2 + \frac{dr^2}{\left(1-\frac{b^2}{r^2}\right)} + r^2\,d\phi^2. \tag{A.2}$$

It is worth mentioning that for the chosen function $A(r)$, the space-time (A.2) satisfies all the requirement of a wormhole geometry, namely the flare-out condition, asymptotic flatness etc.. Now, we redefine the radial r and azimuthal coordinates ϕ by the substitution $r \to r' = \frac{r}{\alpha}$ and $\phi \to \phi' = \alpha\,\phi$, where $\alpha < 1$ in the above metric (A.2), one will obtain

$$ds^2 = -dt^2 + \frac{\alpha^{-2}\,dr^2}{\left(1-\frac{\alpha^2\,b^2}{r^2}\right)} + r^2\,d\phi^2. \tag{A.3}$$

The above space-time is called a circularly symmetric and static $(1+2)$-dimensional wormhole metric with cosmic strings.

Finally transforming to a new coordinate via $r^2 = \alpha^2\,(x^2+b^2)$ which covers the whole wormhole region, one will find the same space-time (3) given by

$$ds^2 = -dt^2 + dx^2 + \alpha^2\,(x^2+b^2)\,d\phi^2. \tag{A.4}$$

APPENDIX B: THE CONFLUENT HEUN EQUATION

he standard form of the confluent Heun equation is [49–52]

$$H''(x) + \left[\zeta + \frac{\beta+1}{x} + \frac{\gamma+1}{x-1}\right] H'(x) + \left[\frac{\mu}{x} + \frac{\nu}{x-1}\right] H(x) = 0, \tag{B.1}$$

where $H(x) = H_c(\zeta,\beta,\gamma,\delta,\eta;x)$ is the confluent Heun function. The parameters μ and ν given in the last term of Eq. (B.1) are defined as

$$\mu = \frac{1}{2}\,(\zeta + \zeta\,\beta - \beta - \beta\,\gamma - \gamma) - \eta, \quad \nu = \frac{1}{2}\,(\zeta + \zeta\,\gamma + \beta + \beta\,\gamma + \gamma) + \delta + \eta, \tag{B.2}$$

By using the Frobenius method [53], one will obtain a polynomial solution to the confluent Heun equation. Let us write the confluent Heun function as a power series around the origin,

$$H(x) = \sum_{i=0}^{\infty} d_i\,x^i, \tag{B.3}$$

where d_i are the coefficients.

Thereby, substituting this power series in the Eq. (B.1), one will obtain the coefficient

$$d_1 = -\frac{\mu}{\beta+1}\,d_0, \tag{B.4}$$

with the following recurrence relation

$$d_{k+2}=\frac{1}{(k+2)(k+2+\beta)}\Big[\Big\{(k+1)(k+2+\beta+\gamma-\zeta)-\mu\Big\}\,d_{k+1}+(\zeta\,k+\mu+\nu)\,d_k\Big]. \tag{B.5}$$

Therefore, from the Eq. (B.5), the confluent Heun series becomes a polynomial of degree n when we impose two conditions:

$$d_{n+1}=0, \quad \delta=-\zeta\left[n+\frac{1}{2}\,(2+\beta+\gamma)\right], \tag{B.6}$$

where $n=1,2,3,.....$ But, we don't know whether a closed expression for the energy eigenvalue will exists or not.
